# A Meta-Analysis and Systematic Review of Community-Based Intimate Partner Violence Interventions in India

**DOI:** 10.3390/ijerph20075277

**Published:** 2023-03-27

**Authors:** Mona Mittal, Anna Paden McCormick, Manjushree Palit, Nicole Trabold, Chelsea Spencer

**Affiliations:** 1 Department of Family Science, University of Maryland School of Public Health, College Park, MD 20742, USA; 2 Jindal School of Psychology and Counseling, Jindal Global University, Sonipat 131001, India; 3 College of Health Science and Technology, Rochester Institute of Technology, Rochester, NY 14623, USA; 4 Department of Applied Human Sciences, Kansas State University, Manhattan, KS 66506, USA; cspencer@ksu.edu

**Keywords:** India, intimate partner violence, IPV, community-based, intervention, meta-analysis, systematic review, family science

## Abstract

Intimate partner violence (IPV) in India remains an entrenched and prevalent public health issue. Despite ample evidence of the widespread problem of IPV in India and associated mental and physical morbidities, far less is known about intervention models to reduce IPV in India. The aims of this meta-analysis and systematic review are to assess the effectiveness of community-based interventions to reduce IPV in India and to provide a narrative synthesis of these intervention approaches. A total of 9 databases were searched to identify peer-reviewed, English-language articles published between January 2000 and September 2022. The search identified 10 studies that met study inclusion criteria, including 3 randomized control trials, 4 quasi-experimental, 2 pre/post, and 1 time-series evaluation. Eight studies were included in the meta-analysis. There was notable variation in the interventions and approaches employed to reduce IPV and varying measurement of IPV outcomes. The results of the meta-analysis show that participating in community-based IPV interventions produced a significant reduction in IPV among women. When considering different types of IPV, study participants were less likely to report physical and psychological IPV victimization. In addition, participants were also less likely to report approving of IPV after participating in community-based IPV interventions. Community-based interventions and research addressing IPV are still evolving in India. Missing descriptions of theoretical frameworks, sampling, intervention design, and inadequately reported effectiveness of intervention (both quantitative and qualitative reporting) need to be addressed. Moreover, long-term evaluations of the pilot interventions are needed to provide a clear picture of the long-term effectiveness, sustainability, and replicability of the community-based IPV interventions. The findings have implications for researchers, practitioners (community health workers, clinicians, and social workers), and policymakers keen on IPV reduction in India and globally.

## 1. Introduction

Intimate partner violence (IPV), sometimes called domestic violence (DV), continues to be a significant global public health issue. The World Health Organization defines IPV as any behavior by an intimate or ex-partner that causes physical, sexual, or psychological harm, including physical aggression, sexual coercion, psychological abuse, and controlling behaviors [1]. A recent report by the WHO on the prevalence estimates of lifetime IPV among women documents that among ever-married/partnered women aged 15–49 years, the lifetime and past 12-month prevalence of physical and/or sexual IPV was much higher in Southern Asia (35%) and (19%) compared with countries in North America (25%) and (6%), respectively [1]. A recent systematic review on domestic violence in India estimated that approximately 41% and 30% of women in India experience lifetime and past 12-month domestic violence, respectively [2]. This corresponds to 4 in 10 Indian women experiencing lifetime domestic violence and 3 in 10 experiencing domestic violence in the past year. Among ever-married women between the ages of 15–49 who have experienced IPV, 29.2% report physical violence, 6.7% sexual, and 32.8% report emotional violence [3]. While there are regional variations, acceptance of marital violence continues to be very high; 45% of women and 44% of men between the ages of 15–49, who participated in the National Family Health Survey, 2019–2021 (NFHS-5), endorsed that a man was justified to beat his wife [4].

There is a substantial body of literature on the deleterious impacts of IPV on women. IPV is the leading cause of injury among women ranging from bruises, fractures, traumatic brain injuries, to chronic disabilities [5,6,7]. Women dealing with IPV experience a host of negative short- and long-term health consequences. Research shows that abused women experience higher rates of physical health problems such as cardiovascular disease and hypertension [8,9], sexually transmitted infections [10,11,12], and HIV [13,14]. IPV is also associated with the development of mental health issues such as depression, PTSD, anxiety, and suicidal ideation [15,16,17]. Women dealing with IPV experience adverse reproductive health consequences such as unintended pregnancies, abortions, miscarriages, and labor and delivery complications [18,19].

In the last five decades, research on risk factors for IPV has exploded. While there are many universal risk factors for IPV worldwide [1], increasingly researchers have documented the influence of culture on IPV experiences among women [20,21,22]. Several studies have explored risk factors for IPV in India. The literature shows that there are similarities and differences among risk factors for IPV in the Indian context compared with studies from other parts of the world. Studies suggest that IPV is associated with childhood abuse, lower socio-economic status, low education, alcohol misuse by the male partner, high social acceptance of violence against women [23,24,25,26,27], young age at first marriage [25]; and male depression and marital dissatisfaction [24]. There are mixed findings about women’s employment, with some studies suggesting that women’s unemployment is a risk factor for IPV [23] and others suggesting otherwise [26,28]. Some culturally influenced risk factors include issues related to dowry [29], infertility [30], male child preference [31], and reproductive decision making [32]. While assessing the fit of the WHO definition of IPV for the Indian context, Kalokhe and colleagues (2016) also highlighted that in India it is very important to consider violence perpetrated by the husband along with other members of his family. This finding has been highlighted by other researchers as well [33,34].

Despite the high prevalence of IPV in India and a growing understanding of its determinants and detrimental health impacts, there is a small body of empirically validated IPV interventions in India. The goals of this meta-analysis and systematic review are to summarize and evaluate the evidence on community-based interventions for women at risk for IPV or experiencing IPV in India. We will identify and explain theoretical frameworks and practice models that underlie these interventions to help clinicians, public health practitioners, and researchers seeking to develop and implement programs to reduce IPV in India.

## 2. Materials and Methods

### 2.1. Identifying Relevant Studies

A systematic review of studies on IPV interventions in India was conducted using guidelines for meta-analysis [35]. A total of 9 databases (Pubmed, PsycINFO, Google Scholar, 3ie, Proquest, EBSCO, Social Services Abstracts, ERIC, and Proquest Dissertation and Theses) were searched to identify studies in peer-reviewed journals, dissertations, and theses that were published between the years 2000–2022. Search terms used to identify studies were related to couples (*intimate partner* or *couple* or *relationship* or *spouse* or *marital* or *married*), relationship conflict/violence (*violent* or *aggress* or *abuse* or *harm* or *maltreat* or *batter* or *victim* or *perpetrator* or *conflict* or *domestic violence* or *gender-based violence* or *sexual coercion*), country (*India*), and interventions (*intervention* OR *prevent* or *trial* or *program* or *response* or *service* or *package*). The 2020 PRISMA checklist was followed for this study [36].

### 2.2. Study Selection

Eligible studies comprised any primarily quantitative design that evaluated a community-based intervention in India as long as it targeted and included an assessment of physical, sexual, or psychological IPV as a primary outcome. For this review, community-based interventions were defined as interventions where in an entire community received an intervention or where participants for the intervention were recruited via community-based organizations, community groups, or word of mouth within the community. Additional inclusion criteria included: (a) pre-test and post-test scores related to the efficacy/effectiveness of the community-based IPV intervention in order to calculate an effect, (b) report of primary data, and (c) studies published in English regardless of the nationality of the authorship team. Studies included in the meta-analysis reported sample size and effect size of one or more IPV outcomes. 

**Figure 1:**
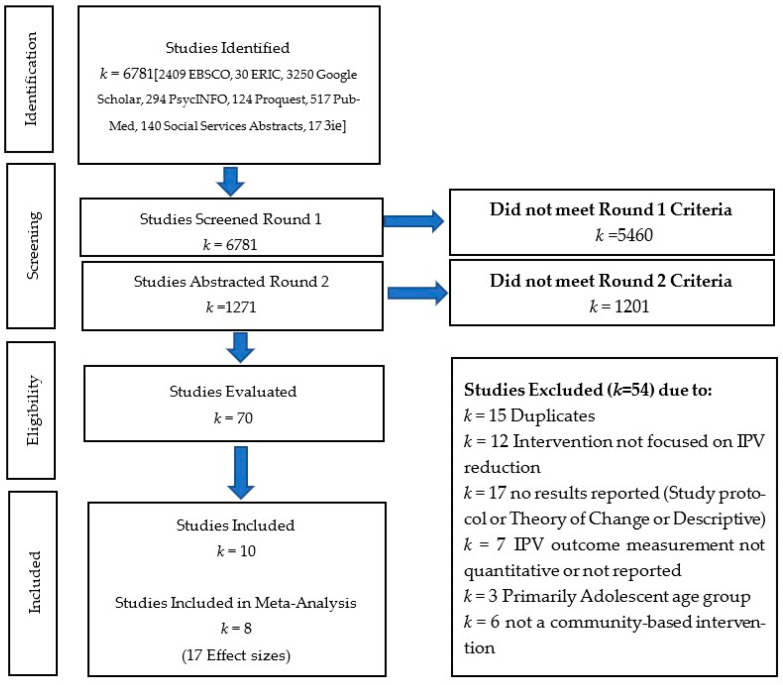
PRISMA flowchart of included studies.

A total of 6781 articles were identified during the initial key terms search. The ERIC search yielded 30 articles, PsycINFO 294, Proquest 124, Social Services Abstracts 140, Proquest Dissertation and Thesis 0, Pubmed 517, 3ie 17, Google Scholar 3250, and EBSCO 2409. Of these initially identified round 1 articles, 5460 (80.5%) were excluded as it was clear from the title and/or abstract that they did not meet the eligibility criteria. During round 2, 1271 articles were carefully reviewed. If eligibility was unclear from the abstract of the article, then the full text was reviewed. This process led to the exclusion of 1201 articles, and a detailed review of 70 articles. Teams of two reviewers (MM and APM, APM and MS, APM and MP, and MM and MP) screened the title and abstract of each record and eventually the full text. Of the 70 studies, 17 studies were either published study protocols, theory of change, or descriptions of interventions with no results being reported. Furthermore, 7 studies either did not report quantitative measurement of IPV data or report any IPV data, 15 were excluded because they were duplicates, 12 studies did not test IPV as a primary outcome of their intervention, 6 studies did not evaluate a community-based IPV intervention, and 3 studies focused only on adolescents. This systematic review includes 10 articles, 8 of which provided 17 usable effect sizes and were included in the meta-analysis. See Figure 1 for the PRISMA flowchart of included studies in the analysis. 

### 2.3. Search Results

Ten publications met the study inclusion criteria. All of them were included in the systematic review and eight in the meta-analysis. Of the 10 studies included in the systematic review, all but 3 employed an experimental design. Three studies were randomized control trials; one randomized couples to one of three arms and two were randomized at the cluster level (village or community). Four studies were quasi-experimental, two were pre/post evaluations, and one study used monitoring data to document incidents of violence over a five-year period [37,38,39,40,41,42,43,44,45,46]. These evaluation designs assessed two couples-based interventions, three individually based interventions with women, and five multi-level interventions seeking to address individual and/or couples-level behavior as well as social norms and structures (see Table 1 for details). A total of 17 unique effect sizes were analyzed to examine 5 IPV-related outcomes.

### 2.4. Data Extraction

Researchers systematically extracted the following information from the eligible studies: authors, year of publication, intervention type, target population and sample size, setting, description of intervention including duration, primary and secondary outcomes being evaluated, and statistical information required for calculating effect sizes. Information extracted was reviewed and cross-coded by the study team for accuracy. Synthesis tables were created to review intervention designs, outcome measurements, and identify patterns of effectiveness.

### 2.5. Data Analysis Plan

Effect sizes obtained from the included studies were entered and analyzed using comprehensive meta-analysis 3.0 [47]. A random-effects approach was used when analyzing the data. A random-effects approach allows for increased generalizability of the findings, as it accounts for population differences between studies [35]. For each outcome found in at least 2 studies [48], an aggregate effect size (unadjusted odds ratios) and 95% confidence interval for that outcome was calculated based on the pre-test and post-test data of each study. The following outcome variables were identified for the study: female IPV victimization (all types), female physical IPV victimization, female sexual IPV victimization, female psychological IPV victimization, and approval of IPV. For significant results found in at least three studies, a fail-safe *n* was calculated to ensure that the results were robust against potential publication bias [49]. The fail-safe *n* provides the number of insignificant studies that would be needed to make the current insignificant.

## 3. Results

### 3.1. Narrative Synthesis

#### 3.1.1. Couple-Based Interventions

Community-based interventions with couples were efficacious in reducing IPV and addressing risk factors for IPV in the Indian context [39,41]. These studies utilized an RCT or quasi-experimental design and recruited couples through outreach at women’s groups meetings, community health camps, word of mouth, and/or snowball sampling [39,41]. Hartmann and colleagues (2021) tested an integrated intervention aimed at male alcohol use and IPV reduction. Both studies measured IPV with abridged or modified versions of the Indian Family Violence and Control Scale (IFVCS). Hartmann et al. (2021) omitted the sexual violence domain from the IFVCS after consultation with their community partner but measured the remaining IPV domains. Both the studies measured alcohol use [39,41]. In addition to measuring IPV and alcohol use, Kalokhe et al. (2021) assessed mental health of the female participants as a primary outcome of the study. These interventions were much shorter in duration (4–6 weeks) compared with all but one of the community-based interventions in this review that range in duration from 1–5 years [39,41]. One study recruited newly married couples as a method of primary prevention of IPV [41], and the other recruited couples where the male partner was reported to have a drinking problem [39]. The Gya Bharari Ekatra intervention utilized peer educators to provide psychoeducation and skills to newly married groups of 3–5 couples on relationship quality, resilience, communication, conflict negotiation, self-esteem, sexual health, and IPV social norms [41]. The other study utilized lay counselors with prior social work experience and conducted a three-arm trial [39]. The three arms included a control condition group, an incentive-only for not drinking group, and a behavioral couples therapy (BCT) and an incentive for not drinking group. Of note, at the end of the 1-month BCT intervention utilizing couples counseling and an incentive, a statistically significant 9.9 point drop (95% CI −15.3, −4.5; *p* < 0.001) in overall violence score was reported, and at the 4-month follow-up, a statistically significant 13.3 point drop (95% CI −19.0, −7.6; *p* < 0.001) was reported as compared to the control arm [39]. Reductions in overall violence score were achieved in the incentive-only group; however, the reduction in mean violence score for the BCT couples counseling plus incentive arm was 6.2 points lower at the 4-month follow-up than the incentives-only group [39]. The BCT couples counseling and incentive group also reported a statistically significant drop in the proportion of negative breath alcohol tests compared with the control group [39]. The 6-week intervention with newly married couples reported fewer incidents of psychological abuse in the intervention group compared with the control group participants at the 3-month follow-up, however, this result is not statistically significant [41].

Both interventions were challenged by participants missing sessions and small sample size (*n* = 33–60 couples); however, high retention (95–100%) and high fidelity (85–95%) was achieved by both interventions [39,41]. Both interventions provided promising evidence, especially given the short period of intervention. Newly married couples may not be at high risk for physical or sexual violence. Therefore, a larger sample size and longer endline measurement may be needed to see the effect of the intervention on physical or sexual IPV.

#### 3.1.2. Individually Focused Interventions with Women

Individually focused interventions with women that sought to reduce IPV showed mixed but promising evidence [38,43,45]. Three different study designs were used to evaluate the three interventions with women: RCT, quasi-experimental, and pre/post evaluation [38,43,45]. Two of the three interventions were integrated IPV and HIV risk reduction interventions that recruited women married to men abusing alcohol [38,45]. Both the interventions included a sexual health module designed to synergize with problem solving, marital communication, and conflict resolution skills modules. While both of these interventions had a group component, the duration and the approach of the interventions were markedly different. The Saggurti et al. (2014) intervention was guided by social cognitive theory (SCT) and the theory of gender and power (TGP) and was delivered over 6–9 weeks by a trained master’s-level counselor. It included four individual counseling sessions interspersed with two group sessions. The Cottler et al. (2010) study was 2 months in duration and was delivered by trained peers in a group format.

The authors of the Saggruti et al. (2014) study used 3 questions, 1 each, to measure (a) marital conflict (Did you and your husband have any argument in the past 3 months?), (b) physical and sexual violence (Have you and your husband had an argument or fight where he physically or sexually hurt you in the past 3 months?), and (c) sexual coercion (Was there any coercion or pressure on you to have sex the last time you had sex with your husband?). The Cottler et al. (2011) measured IPV using a violence exposure questionnaire that assessed women’s experience of physical, emotional, and sexual abuse and perpetration of physical violence. Considerable differences in measurement of the IPV-related outcomes in these studies impede direct comparison. The Cottler et al. (2011) study reported a small but statistically significant reduction in IPV victimization, while the Saggruti et al. (2014) reported no change in physical and sexual violence. Both studies reported statistically significant reductions in other IPV-related variables. Cottler et al. (2010) reported that women enrolled in the program not only experienced a significant reduction in male partner perpetration of violence, but also were less likely to retaliate and be perpetrators of violence toward their male partner. Correspondingly there was a reduction in the percent of women anticipating a high to moderate chance of being abused, attacked, or forced to have sex [38]. This may have improved participant’s overall wellbeing by reducing daily felt fear and related stress [38]. Saggurti et al. (2014) reported statistically significant reductions in marital conflict and marital sexual coercion.

The third study used a 2-arm (intervention and a wait-listed control group), pre-and post-intervention design, with a 6-month follow-up among college students [43]. The intervention group received a group-based sexual violence intervention program that was focused on reducing sexual violence (experiences of unwanted kissing, unwanted touching, sexual harassment, and rape in the last 3 months) by improving knowledge, promoting positive attitudes, and supporting effective behaviors to prevent sexual violence [43]. This intervention was relatively brief and involved 5, 2 h training sessions over the course of 5 weeks. The evaluation reported no statistically significant reduction in sexual violence victimization, and relationship or bystander behaviors, but improvements in knowledge related to gender, sexual violence, bystander and healthy relationship communication, and bystander attitudes and intentions were reported [43]. Neider et al. (2022) reported a significant and substantial improvement in bystander knowledge (22%) compared with the comparison group, and a modest statistically significant improvement in bystander intentions (5%) among those who received the 10 h intervention and compared with the comparison group [43]. While it was not statistically significant, the authors also reported a 11% increase in bystander intervention behavior, a key socio-cultural variable, this is notable for the relatively short follow-up period of 6 months [43].

All three interventions reported improvements in violence-related outcomes [38,43,45], and one reported a small but statistically significant drop in IPV [38]. Neider et al. (2022) noted that in India the sexual violence intervention for female college students did not result in statistically significant improvements in any of the behavior-related outcomes indicating a need to extend time before the endline measurement, and also to review the intervention design in the context of India.

#### 3.1.3. Multi-Level Interventions

Multi-level community-based interventions to reduce IPV presented promising evidence of an effect on IPV. These interventions applied health promotion programming that included a focus on IPV at the community level and IPV risk reduction programming with high-risk groups (women and sex workers) with the goal of catalyzing sustained change in socio-cultural as well as individual- and familial-level risk factors related to IPV. Multi-level intervention studies were conducted with youth between the ages of 16–24 [37], women across 22 villages [41], sex workers and their communities [40,44], and men who drink alcohol [46]. The Balaji et al. (2011) study with male and female youth age 16–24 and the Nair et al. (2020) study with women assessed the effectiveness of multi-level interventions over 16–18 months. Both studies combined activities at different levels, such as peer or women’s group (ASHA)-delivered group sessions, street plays, gatherings, health information distribution, and individual referrals for counseling [37,42]. Balaji et al. (2011) reported a large statistically significant reduction in sexual violence in urban intervention sites (81% less likely to experience sexual violence (aOR = 0.19 95% CI 0.09–0.41), and large statistically significant reductions in perpetration of physical violence in urban intervention sites (41% less likely to perpetrate physical violence (aOR = 0.59 95% CI 0.40–0.87)) and rural intervention sites (71% less likely to perpetrate physical violence (aOR = 0.29 95% CI 0.15–0.57)). Associated with these decreases, Balaji et al. (2011) also reported statistically significant 38.8% and 60.1% decreases in depression among youth in the urban and rural intervention areas, respectively. Nair et al. (2020) reported a statistically significant reduction in the experience of emotional violence among study participants. At post-test, Nair et al. (2020) reported a 11.1% reduction in emotional abuse from husbands, which provides evidence that the intervention reduced the likelihood of a woman experiencing emotional violence by 45% (aOR = 0.55 (0.43–0.71 *p* < 0.001). In tandem with reduction in emotional abuse by husbands, post-test results also showed a 16% reduction in reported physical violence by family members, which provides evidence that the intervention reduced the likelihood of a woman experiencing physical violence by family members other than the husband by 59% (aOR = 0.41 (0.32–0.53, *p* < 0.001) [41].

Nair et al. (2020) reported a statistically significant increase in the percentage of all intervention participants who believe that violence against women is unacceptable for any reason. At post-test, those who participated in the intervention were 87% more likely to view violence against a woman as “unacceptable” in all situations (aOR 1.87 (1.39–2.52 *p* < 0.001) [41]. Other positive effects such as a substantial improvement in help-seeking behavior, and improvements in knowledge and attitudes about IPV, emotional health, reproductive and sexual health, and substance use were reported [37,42].

Furthermore, 2, 2–5 year long, multi-level interventions with sex workers and communities aimed to reduce violence, including IPV, experienced by sex workers from intimate partners and/or clients [40,44], and increase condom use within intimate relationships of sex workers [40]. These interventions differed in their approach, duration, evaluation design, measurement of intervention effects, and in how data were utilized to implement the intervention. These factors may contribute to differences in intervention outcomes reported by each group [40,44].

The intervention evaluated by Reza Paul et al. (2012) began with intensive recruitment, mobilization, training, and empowerment of sex workers in the community to identify and address root causes of vulnerability and violence. To evaluate the intervention, sex workers were trained to report incidents of physical, emotional, psychological, and verbal violence by boyfriends, clients, police, pimps/agents, and these data were diligently tracked over a 5-year period [44]. The authors reported a fundamental shift in the way intimate partners, police, and community members treated and viewed sex workers in the intervention area [44]. This 5-year intervention with sex workers and communities reported a remarkable reduction (84%) in all violence, including IPV from boyfriends and physical and sexual abuse from clients, pimps, and police as well as associated coercion [44]. This intervention and evaluation involved consistent and prolonged monitoring of violence at the community level by sex workers demonstrating how to utilize monitoring data to empower implementers, beneficiaries, and achieve the goals of the intervention [44]. However, the lack of information about the evaluation design, data collection, and measurement of IPV events prevents a clear understanding of the outcomes and replication of the methodology. For these reasons, we could not include this study in this meta-analysis.

The second study that focused on sex workers, intimate partners, and their community used a cluster-randomized trial and was 27 months long. Study results indicated a statistically significant reduction in acceptance of IPV (38% reduction in intervention groups (aOR = 0.62, 95% CI 0.40–0.94, *p* = 0.025), and statistically significant increases in solidarity of sex workers around issues of IPV and awareness of self-protection strategies [40]. A reduction in IPV was seen in the intervention group; however, it was not statistically significant [40]. Like Reza-Paul et al. (2012), this intervention also sought to train and empower sex workers; however, the sex workers did not play as central a role in identifying structural causes of violence, developing and advocating for specific types of changes, or tracking incidents of violence [40]. These intervention design differences and differences in the duration of the interventions (27 months vs. 5 years) and markedly different evaluation methodologies may contribute to the different outcomes reported [40,44].

The last multi-level, 3 year, community-based intervention aimed to reduce IPV by addressing sexual risk reduction, problematic drinking, and improving marital relationships among married men ages 21–40 [46]. The intervention used several approaches ranging from street plays, community meetings, poster and banner presentations, videos and movies, printed materials, and interpersonal communication between men in the community and study staff. No statistically significant reduction in spousal violence (IPV) was seen among men longitudinally tracked. However, statistically significant improvements in attitudes about gender equity and reductions in extramarital sex were achieved [46]. Results are reported based on drinking behavior (stratification) in order to see the effect of the intervention. Except for those who were not drinking at baseline but were drinking at endline, there were improvements in IPV-related variables of extramarital sex (significant drop), and gender-equitable attitudes (small significant improvement) [46].

### 3.2. Outcomes of Community-Based IPV Interventions

Individuals reported a lower likelihood of female IPV victimization after participating in community programs compared with before they participated in the program (*OR* = 0.61, *95% CI* = 0.48–0.79, *p* < 0.001) [See Table 2]. This finding was robust against potential publication bias (fail-safe *n* = 151). When looking at different types of IPV, it was found that participants were less likely to report female physical IPV victimization (*OR* = 0.50, *95% CI* = 0.31–0.80, *p* = 0.004) and female psychological IPV victimization (*OR* = 0.42, *95% CI* = 0.24–0.75, *p* = 0.003) after participating in community programming. Participants were also less likely to report approving of IPV after participating in the community program (*OR* = 0.63, *95% CI* = 0.44–0.89, *p* = 0.009). Participants did not report significantly lower levels of female sexual IPV after participation in community programs (*OR* = 0.58, *95% CI* = 0.27–1.25, *p* = 0.164). See the Supplementary Materials for forest plots of the meta-analyses (see Figures S1–S5).

## 4. Discussion

To the best of our knowledge, this meta-analysis and systematic review is the first to critically examine and synthesize studies evaluating community-based IPV interventions in India. Given the robust body of evidence documenting the prevalence and burden of IPV in India, it is remarkable and concerning that this review revealed only 10 community-based IPV interventions in peer-reviewed journals. Of these, only eight reported data in a manner conducive for inclusion in the meta-analysis. This is a fraction of the 137 peer-reviewed articles, published between 2004–2015, reporting on the prevalence of IPV and IPV-associated risk factors in India as identified in a recent systematic review of literature [2]. Despite this, results from the meta-analysis provide compelling evidence of the impact of these interventions in reducing the odds of IPV victimization among women by 39%. Further, when considering different types of IPV, while participants did not experience a significant reduction in sexual IPV, participation in these community-based interventions reduced the odds of experiencing physical and psychological IPV by 50% and 58%, respectively. The results also show a reduction in acceptance of IPV among study participants by 37%.

Interventions in this review varied regarding goals, level of the socio-ecological model that they targeted (individual, couple, and community), delivery, frequency, and duration. However, the individually focused and couple-based interventions were short-term, ranging from 4–6 sessions, and were either delivered in groups or a combination of groups and individual couple sessions. Few of the authors discussed the theoretical models that informed their interventions. Of the studies that mentioned a specific theory of change as the basis for their intervention, one used interdependence theory [41], one used BCT and contingency management [39], and one used SCT and TGP [45]. Intervention program content included healthy relationships, communication, conflict negotiation, sexual health-related knowledge, motivation, and skills, reproductive health and wellness, alcohol use, gender, and violence. Most of these interventions were delivered by trained peers or lay counselors. Only one study was delivered by trained master’s-level counselors [45]. The multi-level, community-based interventions were much longer in duration, ranging from 12 months to 5 years [37,40,42,44,46]. These interventions involved community-wide mobilization activities such as street plays, gatherings, health information distribution, and posters, and some also included peer-delivered group sessions and individual referrals for counseling [37,40,42,44,45]. The multi-level interventions were facilitated by either trained peers, ASHA workers, program/agency staff, or a combination of the two.

In addition to a diverse range of intervention designs and content, there was much variation in the measures used to assess IPV outcomes. These ranged from a structured violence exposure questionnaire to single-item measures. Not all the studies assessed for changes in all three major domains of physical, psychological, and sexual IPV. Only two studies used a modified version of the Indian Family Violence and Control Scale, a recent measure that was developed and validated for use in India [39,41]. Future studies should use standardized, accepted, and culturally validated measures for IPV to facilitate cross-study comparisons of intervention efficacy and effectiveness.

The heterogeneity of interventions, theoretical models, and measurement of IPV as identified in this review impede direct comparison of the interventions to identify which interventions are more effective in reducing IPV victimization and its acceptance or related mechanisms of change (see Table 3). However, three important features emerged (see Table 4). First, the individual-focused and couple-based interventions, despite having the shortest durations (1–2 months), showed significant evidence of intervention efficacy in reducing violence [39], as well as marital conflict and marital sexual coercion [45]. The intervention evaluated by Kalokhe et al. (2021) also presented promising trends in intervention beneficiaries with non-significant reduction in psychological abuse of women and improvement in female mental health. These interventions also present evidence of improvement in husbands’ alcohol abuse [39]. As noted by Saggurti et al. (2014), the “results show that a low-intensity, low cost intervention” may powerfully empower women with skills to seek formal and informal support and resources to address their marital difficulties.

Second, interventions that addressed distal and proximal factors driving IPV by intensively incorporating and consulting with community members and study participants to understand the violence-specific structural, community, cultural, familial, relational, and/or individual factors achieved greater reductions in IPV and violence against women more broadly along with significantly large improvements in attitudes about violence against women [42,44]. Nair et al. (2020) reported a large statistically significant reduction in the percent of physical violence by family members other than the husband (16% reduction at post-test) through a phased intervention that focused primarily on addressing and changing social norms. This intervention incorporated information gathered from women and community members in one phase to empower the same women and community members to “identify locally feasible strategies” to address violence against women including IPV in the subsequent phases [42]. The intervention evaluated by Reza-Paul et al. (2012) utilized a similar empowerment-based approach to identify and address structural drivers of violence, thereby making drastic reductions in violence experienced by sex workers.

Third, interventions with young adults reported reductions in experiencing sexual abuse, perpetration of physical violence, and substance use [37]. They also reported improvements in mental health, knowledge, and attitudes about gender, reproductive and sexual health, sexual violence, and healthy relationships [42]. Balaji et al. (2011) reported a statistically significant 72.2% proportional drop in experience of sexual abuse among urban community intervention sites (5.4% at baseline to 1.5% at endline). Participation in the intervention in urban communities reduced the likelihood of experiencing sexual violence by 81% (OR = 0.19 (95% CI 0.09–0.41) [37]. Balaji et al. (2011) also reported a statistically significant 61% (rural) or 38.8% (urban) proportionate reduction in probable depression (score of 4 or above on the general health questionnaire) among young adults in intervention sites, and a 58.6% (rural) and 49.5% (urban) proportionate reduction in perpetration of physical violence among young adults in intervention sites. An intervention with newly married couples, a similar age group, reported similar, yet non-statistically significant, trends including improvement in overall mental health of female participants and fewer incidents of psychological abuse in the intervention group [41]. This age group may present an optimal population for primary-prevention IPV interventions, as information on healthy relationships, gender, reproductive and sexual health, and substance use may be timely, of great interest, and usefulness [43].

### Implications for Research and Practice

The studies included in this review had several methodological limitations. Sample sizes tended to be small, particularly for the individually focused and couple-based studies. The authors of one such study noted that the IPV outcomes were insufficiently powered to detect change [45]. Guides for calculating sample size for IPV interventions may be a powerful resource to enable the generation of better quality IPV intervention evaluation data. Most of these studies were pilot studies focused on feasibility and acceptability and had not been replicated with larger samples or in different settings. IPV in India is driven by context-specific factors and therefore needs context-sensitive intervention design to be effective, and this likely means repeated evaluation to improve IPV interventions design and implementation practices to ensure effectiveness and impact. Future studies should assess the longer-term impact of these interventions. Researchers should also consider reporting sub-group analysis to further our understanding of intervention effectiveness across different groups.

There are data about the prevalence of IPV in India, and the populations most impacted, however, practitioners may not readily have access to this information when designing IPV reduction programs [2]. Programs that effectively reduce IPV in other countries and contexts will not necessarily have the same effect in Indian communities. Neider et al. (2022) noted that in India their intervention did not change participants attitudes toward gender stereotypes, ability to communicate needs and feelings in relationships, or decrease acceptance of rape myths, whereas the intervention was found to be effective in the USA in these areas. Interventions in this review were primarily designed to improve sexual and reproductive health [37,38,45], reduce alcohol abuse [39,46], or were integrated interventions aimed at addressing IPV and alcohol abuse [38,39,46], or IPV and HIV risk [38,40,44,45]. Most of the studies included in this paper also addressed acceptance of gender-based norms for IPV and violence against women, and included knowledge enhancing and skill-based exercises focused on health, relationships, communication, conflict management, decision making and problem solving [37,40,41,42,43,44,45,46]. However, not many addressed other specific drivers of IPV victimization and perpetration that have been identified within the Indian context. Other determinants of IPV victimization and perpetration, including childhood abuse, early marriage, male child preference, male depression, sexual communication, marital dissatisfaction, family violence, and financial stress should be incorporated into IPV reduction interventions in India.

Furthermore, given the multifactorial and often invisible drivers of IPV, quantitative measures should not be regarded as a gold standard, but rather one of the two core strands of information that evaluations gather. Of the 10 studies reviewed, only 4 incorporated qualitative data collection as part of the overall intervention evaluation [37,41,44,46], and these data are interwoven in the interpretation and discussion of the quantitative findings in 2 out of the 4 studies [44,46]. Moreover, several studies provided limited or poor descriptions of intervention design and theoretical approaches impeding the full understanding of how the intervention led to the observed changes at endline. To increase the likelihood of clearly understanding intervention effectiveness or lack of effectiveness, and to empower practitioners and communities, resources and tools for basic qualitative data collection should be developed and disseminated. Providing more detailed information about the interventions and presenting process evaluations will also aid in intervention replication and thereby strengthen the evidence base of IPV interventions in India. Questions generated by these studies are powerful and should be considered by future evaluators (see Table 5).

## 5. Conclusions

Limited evidence of the efficacy and effectiveness of community-based IPV reduction interventions has impeded the forward momentum to address this complex issue in India. Despite the limited number of studies available and gaps in the evidence, these evaluations are promising. Future efforts should build on the current evidence base, consider leveraging the existing community health (CHWs) networks and women’s groups, address additional IPV predictors for IPV victimization and perpetration in India, and use culturally tailored assessments for IPV. Researchers and practitioners will also benefit greatly from using a community-based participatory framework when designing interventions and their evaluations.

## Figures and Tables

**Table 1:** Evaluations of community-based IPV interventions in India.

Table One: Evaluations of Community-Based IPV Interventions in India							
Study	Condition of Intervention	Intervention Description	Study Design	Duration	Sample	Age of Participants	Primary Outcome
Interventions with Couples							
Hartmann et al. 2021 [39]	Interventions with couples	Study with 3 arms: control, 4 weekly cognitive behavioral counseling sessions with couples, and 4 weekly cognitive behavioral counseling sessions with couples plus incentive to not drink	3-arm randomized controlled trial	1 month	60 couples from a large city, 20 per arm	Men 27–52 and women 18–42 years of age	Female-reported IPV victimization and breath alcohol concentration among participants (both partners)
Kalokhe et al. 2021 [41]	Interventions with couples	Assign peer educators to groups of 3–5 couples to address relationship quality, resilience, communication, conflict negotiation, self-esteem, sexual health and communication, and norms around IPV.	Quasi-experimental	6 weeks	40 newly married couples residing in slum communities surrounding a major city, 20 per arm	Men averaging 26.4 and women averaging 21.6 years of age	Female-reported IPV victimization (psychological abuse); female mental health
Interventions with Women							
Cottler et al. 2010 [38]	Interventions with women	Women’s groups—Body Wise Intervention—focused on sexual health and behavior	Pre/post evaluation	2 months	100 married women whose husbands reported heavy drinking during a community-wide household survey	18–50 years of age	Female-reported IPV victimization (emotional, sexual, and physical abuse)
Neider et al. 2022 [43]	Interventions with women	Classroom-based training	Quasi- experimental	10 h of training over 5 weeks	254 female university students	17–22 years of age	Female-reported sexual victimization; knowledge and attitudes of gender, healthy relationships and communication, sexual health, and bystander intention
Saggurti et al. 2014 [45]	Interventions with women	Intervention including 4 individual sessions and 2 women’s groups sessions; problem solving sessions with a counselor	2-arm cluster randomized controlled trial	6–9 weeks	220 married women from a low-income community with a history of IPV or male partner heavy drinking	18–40 years of age	Female-reported IPV victimization (physical and sexual abuse); marital conflict; and marital sexual coercion
Multi-Level Interventions							
Balaji et al. 2011 [37]	Multi-level intervention (women, men, sex workers, and youth groups)	Peer educators conduct group sessions; street plays; teacher training program; and a health information campaign at household and community levels	Quasi-experimental	18-month-long intervention	Young adults in 2 urban and 2 rural communities in a state	Young adults 16–24 years of age	Male- and female-reported IPV victimization (physical and sexual abuse), depression, and substance use
Javalkar et al. 2019 [40]	Multi-level intervention (women, men, sex workers, and youth groups)	Sex worker group meetings; peer educator counseling with sex workers; village plays; training of male champions; couples events for sex workers and their IP’s; and a crisis management team	Cluster randomized controlled trial	27 months	547 sex workers from 47 villages in 1 district	Women averaging 34.5 years of age	Female-reported IPV victimization (physical and sexual abuse), acceptance of IPV
Nair et al. 2020 [42]	Multi-level intervention (women, men, and youth groups)	Community mobilization through participatory learning and action—meetings with women’s groups followed by community gatherings	Pre/post evaluation	16 months	679 women at baseline and 861 women at endline from 39 women’s groups across 22 villages in one district	Age range of women not provided	Female-reported IPV victimization (emotional violence from husbands)
Reza-Paul et al. 2012 [44]	Multi-level intervention (women, men, sex workers, and youth groups)	Sex worker-led structural intervention to address root causes of violence against sex workers at the community level: addressed isolation, access to health services, intimidation, harassment, extortion, and rape from men and police, and assault by boyfriends; set up safe spaces, rapid violence response, improved workplace security, increased access to health care/condoms/STI testing, and increased community acceptance	Time-series and incident monitoring	5 years	Sex workers in one community	18 years or older; age range not provided	Female-reported IPV victimization (physical, sexual, emotional/psychological, and verbal) by boyfriends, clients, police, pimps/agents
Schensul et al. 2010 [46]	Multi-level intervention (men and community)	Men’s group meetings with referrals for individual counseling; community-wide health information campaign (street dramas, poster and banner presentations, film showings, and distribution of health communication materials)	Quasi- experimental	3 years	Married men from three communities outside a major Indian city that are daily wage workers, petty traders, and small business owners	21–40 years of age	Male-reported perpetration of violence (physical and verbal) towards spouse; drinking behavior, gender equity attitudes, and extramarital sex

**Table 2:** Meta-analysis results examining IPV outcomes for women and community programming.

Outcome Variable	*k*	*OR*	95% *CI*	Fail-Safe *n*
IPV Victimization (all types)	8	0.61 ***	[0.48, 0.79]	151
Physical IPV Victimization	2	0.50 **	[0.31, 0.80]	--
Psychological IPV Victimization	2	0.42 **	[0.24, 0.75]	--
Sexual IPV Victimization	3	0.58	[0.27, 1.25]	--
Approval of IPV	2	0.63 **	[0.44, 0.89]	--

*k* = number of effect sizes; *OR* = odds ratio; *CI* = confidence interval; ** *p* < 0.01, *** *p* < 0.001; boldface indicates statistical significance.

**Table 3:** Intervention types and notable effects reported.

Intervention Type	Statistically Significant Reduction in Any Type of IPV—Physical, Sexual, Emotional, Verbal?	Reduction in Other IPV-Related Outcomes?	Notable Effects Reported
Couples	Yes, 1 of 2 [39]	Yes, 2 of 2	Statistically significant 10-point drop in violence score among couples experiencing IPV [39]; improvement in female participant mental health in 4–6-week intervention [41].
Women’s	Yes, 1 of 3 [38]	Yes, 3 of 3	Significant reduction in marital conflict and sexual coercion in 6–9-week intervention with an individual plus group component [45].
Multi-Level	Yes, 3 of 5 [37,42,44]	Yes, 5 of 5	Odds of experiencing emotional and physical violence by family other than husband decreased by more than 45% [42]); significant decrease in sexual abuse [72.2% (urban)] and depression [60% (rural) and 38.8% (urban)] among participants 16–24 years of age. [37].

**Table 4:** Summary of findings.

Key Findings
Community-based IPV interventions were multifaceted and addressed distal as well as proximal factors and behaviors (e.g., socio-cultural, structural, familial, interpersonal, and individual) that contribute to IPV.
Participation in community-based IPV interventions reduced the likelihood of IPV victimization among women by 39% (OR = 0.61, *p* < 0.001).
Participation in community-based interventions reduced the likelihood of experiencing physical violence by 50% (OR = 0.50, *p* = 0.004), psychological IPV by 58% (OR = 0.42, *p* = 0.003), and reduced the acceptance of IPV among study participants by 37% (OR = 0.63, *p* = 0.009). The meta-analysis results indicate that individuals did not report experiencing significantly lower levels of sexual IPV after participation in community programs (OR = 0.58, *p* = 0.164). See Table 2 for more detail.
The individually focused and couple-based interventions, despite having the shortest durations (1–2 months), showed significant evidence of intervention efficacy in reducing IPV ([39] as well as marital conflict and marital sexual coercion [45])
Interventions that conducted root-cause analysis with beneficiary populations and then used this information on structural, community, cultural, familial, relational, and/or individually based drivers of IPV to guide intervention design and activities, achieved greater reductions in violence, including IPV, and significantly large improvements in attitudes about violence against women [37,42,44]
Interventions with young adults reported reductions in sexual abuse, perpetration of physical violence, and substance use, and improvements in mental health, knowledge and attitudes about gender, reproductive and sexual health, sexual violence, and healthy relationships [37,41,43]. This age group may present an optimal population for primary-prevention IPV interventions, as information on healthy relationships, gender, reproductive and sexual health, and substance use may be timely—of great interest and usefulness [37,41,43]

**Table 5:** Summary of questions for future research.

Future Research Questions
Are victimization rates more likely to drop if women are the primary beneficiaries and targets of IPV intervention, compared with men or couples or communities?
Is there an ideal age range or life stage when IPV interventions are most effective in preventing an individual from IPV perpetration or victimization and/or improving health outcomes?
What types of interventions are more likely to affect sustained reduction in IPV?
Are IPV interventions more efficacious and/or able to effect greater change if combined with interventions that address violence against women from family members?

## Data Availability

No new data were created or analyzed in this study. Data sharing is not applicable to this article.

## Supplementary Materials

The following supporting information can be downloaded at: https://www.mdpi.com/article/10.3390/ijerph20075277/s1, Figure S1: Forest plot for IPV Victimization (all types), Figure S2: Forest plot for Physical IPV Victimization, Figure S3: Forest plot for Psychological IPV Victimization, Figure S4: Forest plot for Sexual IPV Victimization, Figure S5: Forest plot for Approval of IPV.
