# Supplementary material for: A Meta-Analysis and Systematic Review of Community-Based Intimate Partner Violence Interventions in India
Source: Int J Environ Res Public Health. 2023 Mar 27;20(7):5277. doi: 10.3390/ijerph20075277 (PMC10093839; doi:10.3390/ijerph20075277)
Supplement: Supplementary file 1 [file ijerph-20-05277-s001.zip › ijerph-2204482-supplementary.pdf]

International Journal of
Environmental Research and Public Health

MDPI

Study name	Odds ratio	Lower limit	Upper limit	Z-Value	p-Value
Balaji, et al., 2011	0.520	0.407	0.664	-5.242	0.000
Cottler et al., 2010	0.483	0.372	0.628	-5.450	0.000
Hartmann et al., 2021	0.374	0.265	0.530	-5.539	0.000
Javalkar et al., 2019	1.125	0.616	2.052	0.383	0.702
Kalokhe et al., 2021	0.643	0.119	3.463	-0.514	0.607
Nair et al., 2020	0.560	0.449	0.698	-5.144	0.000
Neider, et al., 2022	1.179	0.860	1.615	1.024	0.306
Saggurti et al., 2014	0.601	0.475	0.761	-4.229	0.000
	0.614	0.479	0.788	-3.836	0.000

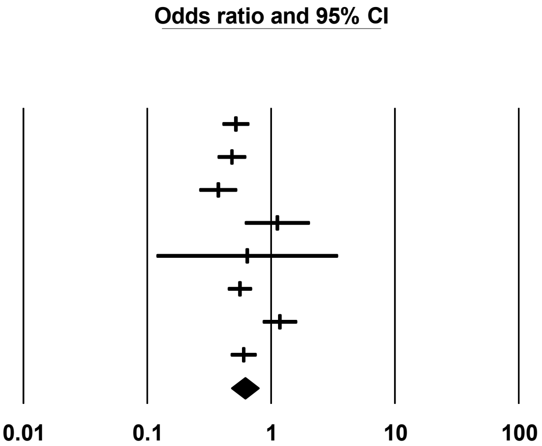

Figure S1. Forest plot for IPV Victimization (all types).

Study name	Odds ratio	Lower limit	Upper limit	Z-Value	p-Value
Balaji, et al., 2011	0.596	0.457	0.777	-3.829	0.000
Hartmann et al., 2021	0.360	0.197	0.660	-3.306	0.001
	0.500	0.313	0.800	-2.891	0.004

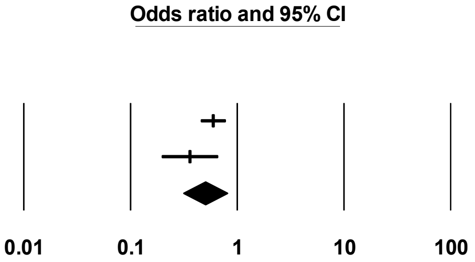

Figure S2. Forest plot for Physical IPV Victimization.

Study name	Odds ratio	Lower limit	Upper limit	Z-Value	p-Value
Hartmann et al., 2021	0.403	0.222	0.732	-2.984	0.003
Kalokhe et al., 2021	0.643	0.119	3.463	-0.514	0.607
	0.425	0.242	0.745	-2.985	0.003

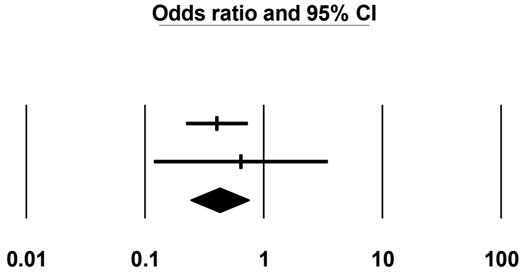

Figure S3. Forest plot for Psychological IPV Victimization.

Study name	Odds ratio	Lower limit	Upper limit	Z-Value	p-Value
Balaji, et al., 2011	0.234	0.123	0.443	-4.454	0.000
Neider, et al., 2022	1.179	0.860	1.615	1.024	0.306
Saggurti et al., 2014	0.612	0.439	0.855	-2.884	0.004
	0.579	0.269	1.249	-1.393	0.164

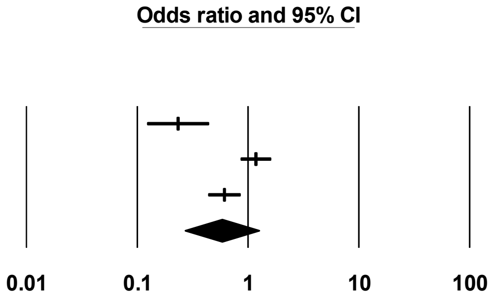

Figure S4. Forest plot for Sexual IPV Victimization.

Study name	Statistics for each study				
	Odds ratio	Lower limit	Upper limit	Z-Value	p-Value
Javalkar et al., 2019	0.767	0.531	1.108	-1.414	0.157
Nair et al., 2020	0.535	0.406	0.704	-4.465	0.000
	0.627	0.441	0.891	-2.605	0.009

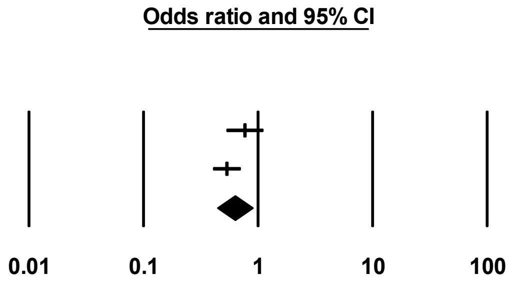

Figure S5. Forest plot for Approval of IPV.
